# Supplementary material for: Genetic Variation in the ASTN2 Locus in Cardiovascular, Metabolic and Psychiatric Traits: Evidence for Pleiotropy Rather Than Shared Biology
Source: Genes (Basel). 2021 Jul 31;12(8):1194. doi: 10.3390/genes12081194 (PMC8391428; doi:10.3390/genes12081194)
Supplement: Supplementary file 1 [file genes-12-01194-s001.zip › genes-1329113-supplementary.pdf]

Genetic variation in the ASTN2 locus in cardiovascular, metabolic and psychiatric traits: evidence for pleiotropy rather than shared biology

Burt *et al*

Supplementary Tables

Supplementary Table 1: All significant assocaitions in the ASTN2 locus.

SNP	BP	A1	BETA	P_sbp	BETA	P_dbp	BETA	P_bmi	BETA	P_neurot	OR	P_anhed	OR	P_moodinstab
9:118957629_AAAG_A	118957629	AAAG					0.045	3.08E-05	-0.021	0.0092	0.99	0.0370		
rs1952977	118957692	G					0.045	2.53E-05	-0.020	0.0122	0.99	0.0398		
rs446783	118957985	T					0.044	3.51E-05	-0.020	0.0121				
rs3789283	118959731	G					0.048	7.76E-06	-0.018	0.0235				
rs449807	118965749	A					0.049	6.31E-06	-0.018	0.0296				
rs452312	118966153	A					0.044	3.59E-05	-0.020	0.0133				
rs405485	118968544	G					0.045	2.56E-05	-0.020	0.0153				
rs10817864	118968579	C					0.045	2.48E-05	-0.019	0.0165				
rs11268296	118969686	TTATTTAAATAG					0.045	3.66E-05	-0.022	0.0066				
9:118970651_CT_C	118970651	C					0.050	4.39E-06	-0.019	0.0184				
rs387747	118977687	G					0.047	1.18E-05	-0.021	0.0100				
rs10817865	118978682	G					0.045	3.25E-05	-0.020	0.0131				
rs418382	118980297	A					0.051	2.27E-06	-0.025	0.0019	0.99	0.0204		
rs398293	118984002	A					0.050	4.07E-06	-0.026	0.0017	0.99	0.0345		
rs620395	118985423	G					0.052	2.14E-06	-0.024	0.0031	0.99	0.0470		
rs415978	118985910	A					0.052	1.75E-06	-0.024	0.0029	0.99	0.0480		
rs432812	118986657	T					0.047	1.44E-05	-0.025	0.0024	0.99	0.0254		
rs373915	118986933	C					0.047	1.47E-05	-0.025	0.0022	0.99	0.0224		
rs400393	118987806	A					0.047	1.46E-05	-0.025	0.0025	0.99	0.0253		
rs384960	118987890	C					0.047	1.34E-05	-0.025	0.0026	0.99	0.0244		
rs438445	118988147	T					0.048	1.25E-05	-0.025	0.0026	0.99	0.0243		
rs399298	118988185	A					0.048	1.25E-05	-0.025	0.0026	0.99	0.0243		
rs398400	118988486	T					0.048	1.15E-05	-0.025	0.0025	0.99	0.0269		
rs407200	118989110	A					0.047	1.38E-05	-0.025	0.0025	0.99	0.0236		
rs432301	118989291	T					0.047	1.37E-05	-0.025	0.0025	0.99	0.0237		
rs13290387	118989926	G					0.047	1.42E-05	-0.025	0.0024	0.99	0.0244		

rs446497	118991117	A					0.047	1.33E-05	-0.025	0.0026	0.99	0.0250		
rs449410	118991707	A					0.048	1.26E-05	-0.024	0.0027	0.99	0.0231		
rs449490	118991762	A					0.047	1.42E-05	-0.025	0.0027	0.99	0.0249		
rs756624	118992381	C					0.048	1.17E-05	-0.025	0.0022	0.99	0.0207		
rs80794	118993074	T					0.050	3.73E-06	-0.024	0.0040	0.99	0.0232		
rs149033263	118993354	GTATATA	0.095	0.0369			0.046	2.94E-05	-0.025	0.0031	0.98	0.0091		
rs428959	118993683	A					0.047	1.39E-05	-0.024	0.0028	0.99	0.0242		
rs426632	118996950	T					0.051	2.80E-06	-0.024	0.0036	0.99	0.0461		
rs10983084	118998517	C					0.046	2.05E-05	-0.024	0.0032	0.99	0.0247		
rs770293683	118999020	TG	0.088	0.0485			0.048	7.60E-06	-0.027	0.0010	0.99	0.0095		
rs12349017	119000973	G					0.048	1.24E-05	-0.024	0.0032	0.99	0.0258		
rs10983085	119001088	G					0.049	8.09E-06	-0.025	0.0028	0.99	0.0265		
rs4837566	119258919	A	0.230	2.60E-05	0.137	1.63E-05								
rs4836732	119266695	C									0.99	0.0293		
rs4837585	119282870	T	-0.189	2.02E-05	-0.119	3.56E-06					1.01	0.0396		
rs77459427	119294799	C	-0.182	4.56E-05	-0.119	3.92E-06					1.01	0.0332	1.01	0.0490
rs4588930	119295104	A	-0.176	7.34E-05	-0.119	3.69E-06					1.01	0.0331		
rs6478243	119296073	C	0.121	0.0077	0.073	0.0058					0.98	0.0095	0.99	0.0316
rs13294352	119296736	T									0.99	0.0269		
rs12379134	119300776	C	-0.134	0.0024	-0.082	0.0013								
rs13283416	119301607	G	0.112	0.0118	0.068	0.0084								
rs1542987	119302317	C	-0.183	4.06E-05	-0.118	4.81E-06								
rs7029693	119308184	T									0.99	0.0305		
rs10671101	119310385	C	-0.162	0.0003	-0.109	2.21E-05					1.01	0.0215		
rs1861881	119312256	T	0.183	0.0001	0.125	4.42E-06								
rs1895062	119313486	G	0.100	0.0247	0.061	0.0176					0.98	0.0062		
rs13290997	119314217	A	-0.159	0.0003	-0.106	3.18E-05					1.02	0.0117	1.01	0.0499
rs1407243	119314851	T	0.110	0.0144	0.062	0.0162					0.98	0.0087		
rs4836750	119315095	T	-0.162	0.0003	-0.107	2.96E-05					1.02	0.0116	1.01	0.0442
rs4836751	119315120	A							0.032	0.0110	1.02	0.0206	1.03	2.52E-05

rs10759848	119323187	A					0.032	0.0095	1.02	0.0194	1.03	3.09E-05
rs1321917	119324929	C	0.100	0.0245	0.059	0.0216			0.98	0.0056		
rs76882529	119325999	C									1.02	0.0049
rs10759849	119328189	T							0.99	0.0116		
rs10983236	119330060	G	-0.168	0.0001	-0.107	2.57E-05			1.01	0.0179		
rs7857286	119332395	T	-0.160	0.0003	-0.105	4.01E-05			1.01	0.0157		
rs10759850	119338483	T							0.99	0.0117		
rs11787627	119341037	A							0.99	0.0135		
rs144805050	119342366	C									1.02	0.0054
rs2208562	119344528	C							0.99	0.0112		
rs7856625	119345083	C							0.99	0.0127		
rs12376892	119348005	T										
rs750422919	119350134	TA							0.99	0.0127		
rs202070966	119350933	A							0.99	0.0161		
rs201971808	119362623	G					0.034	0.0044	1.03	0.0026	1.03	3.22E-05
rs7847553	119363460	T	0.095	0.0349					0.99	0.0169		
rs7874884	119367336	C	0.100	0.0259					0.99	0.0179		
rs2416564	119370679	C	0.095	0.0322	0.057	0.0271			0.99	0.0099		
rs2416565	119370722	C	-0.161	0.0002	-0.106	3.35E-05			1.01	0.0123		
rs10983266	119371620	T	-0.164	0.0002	-0.108	2.09E-05			1.01	0.0130		
rs7871810	119374883	T							0.99	0.0135		
rs7856659	119375255	A	-0.160	0.0003	-0.105	3.69E-05			1.01	0.0123		
rs7863794	119376394	G	-0.159	0.0003	-0.106	3.44E-05			1.01	0.0172		
rs7864410	119376840	T	-0.158	0.0003	-0.104	4.06E-05			1.01	0.0133		
rs10817912	119377433	A	0.097	0.0300	0.059	0.0214			0.99	0.0105		
rs10739470	119377717	T	0.096	0.0308	0.059	0.0231			0.99	0.0112		
rs7872429	119381937	T	-0.161	0.0003	-0.105	3.73E-05			1.02	0.0111		
rs4836788	119385237	G	-0.158	0.0003	-0.104	4.04E-05			1.01	0.0123		
rs4837686	119386789	C	-0.160	0.0003	-0.105	3.61E-05			1.02	0.0110		
rs143358686	119387859	ACCACAACTTATTCTAT	-0.166	0.0002	-0.115	1.03E-05			1.02	0.0067		

rs1885244	119392283	T									0.99	0.0144		
rs1885245	119392413	G									0.99	0.0136		
rs7863462	119409536	A									1.03	0.0152	1.03	0.0052
rs811689	119410756	T	-0.161	0.0003	-0.106	3.24E-05					1.02	0.0119		
9:119412040_CCTT_C	119412040	C							0.036	0.0035	1.02	0.0164	1.03	3.81E-05
rs1661304	119419397	G	-0.138	0.0430					0.036	0.0035	1.02	0.0113	1.03	4.28E-05
rs73530828	119424306	A									1.03	0.0156	1.04	0.0023
rs1661305	119426356	G	-0.137	0.0448	-0.078	0.0493			0.036	0.0035	1.02	0.0125	1.03	4.19E-05
rs1661294	119435891	A	-0.163	0.0002	-0.112	1.19E-05					1.01	0.0131		
rs803896	119447750	A	-0.162	0.0002	-0.111	1.30E-05					1.02	0.0110		
rs803938	119454069	A	-0.140	0.0408	-0.080	0.0437			0.036	0.0040	1.02	0.0123	1.03	3.76E-05
rs803935	119456954	C									0.99	0.0468		
rs803933	119457713	G	-0.135	0.0481					0.037	0.0028	1.02	0.0108	1.03	2.94E-05
rs803932	119458020	C									0.99	0.0436		
rs803931	119458684	G									0.99	0.0466		
rs803930	119458931	G									0.99	0.0496		
9:119459229_TA_T	119459229	T	0.113	0.0198							0.99	0.0498		
rs1661299	119464279	C							0.036	0.0036	1.02	0.0145	1.03	3.91E-05
rs803917	119467660	G									0.99	0.0478		
rs201079720	119479578	CT	-0.126	0.0262					0.031	0.0022			1.03	2.90E-05
rs10983324	119485337	A	0.118	0.0147										
rs35979833	119603326	AG					0.049	0.0014	0.035	0.0022	1.03	4.03E-05	1.02	0.0005
rs2296672	119613820	T					0.037	0.0009	0.036	1.28E-05	1.02	0.0103	1.02	4.15E-05
rs1536428	119616042	A					0.037	0.0009	0.035	2.01E-05	1.02	0.0106	1.02	6.02E-05
rs4837952	119619937	A					-0.043	5.06E-05	-0.017	0.0311			0.98	1.23E-05
rs1952225	119620717	A					-0.043	4.09E-05	-0.017	0.0309	0.99	0.0426	0.98	1.39E-05
rs10759869	119621696	T					0.077	3.60E-06	0.038	0.0024	1.02	0.0102	1.02	0.0020
rs10491574	119622438	T					0.078	2.05E-06	0.037	0.0027	1.02	0.0112	1.02	0.0018
rs13284474	119622710	T					-0.045	2.52E-05	-0.022	0.0064	0.99	0.0189	0.98	7.42E-07
rs7036590	119623110	T					-0.045	1.76E-05	-0.017	0.0283			0.98	7.91E-06

rs4837955	119626482	T					-0.046	1.81E-05	-0.017	0.0378			0.98	2.37E-05
rs13287661	119627075	T					-0.046	1.37E-05	-0.018	0.0274			0.98	2.77E-05
rs7862589	119627987	T					-0.042	8.04E-05	-0.020	0.0111			0.98	8.39E-06
rs4837958	119629708	G					0.045	2.73E-05	0.027	0.0008	1.01	0.0258	1.02	1.44E-05
rs4836881	119629807	G					0.045	2.87E-05	0.026	0.0012	1.01	0.0148	1.02	3.93E-05
rs9696212	119630148	G					0.054	9.05E-06			1.01	0.0486	1.02	0.0020
rs10983386	119631338	T					-0.044	4.07E-05	-0.017	0.0380			0.98	0.0002
9:119632221_AT_A	119632221	AT					0.051	2.95E-05	0.023	0.0124	1.02	0.0068	1.02	0.0022
rs10817947	119633124	A					0.092	2.33E-06						
rs1854027	119634436	T					0.044	6.78E-05	0.028	0.0005	1.02	0.0137	1.02	1.81E-05
rs144850429	120192598	T					0.116	0.0228			1.12	3.52E-05		
rs524440	120307012	T							0.088	1.03E-05			1.04	0.0018
rs60242518	120319115	A	0.171	0.0096	0.166	1.39E-05	0.042	0.0085					1.02	0.0336
rs35769211	120322230	G	0.172	0.0091	0.168	1.10E-05	0.043	0.0074					1.02	0.0304
rs34432054	120324399	T	0.177	0.0071	0.174	4.97E-06	0.041	0.0099					1.01	0.0428
rs17245274	120324763	G	0.176	0.0075	0.173	5.61E-06	0.041	0.0098					1.01	0.0441
rs579017	120326600	T					0.049	0.0255	0.084	3.58E-07			1.03	0.0013
rs568837929	120326945	TA							-0.034	3.57E-05				
rs71507520	120328374	T	0.175	0.0078	0.173	4.97E-06	0.041	0.0095					1.02	0.0339
rs35663424	120332050	T	0.175	0.0079	0.173	5.11E-06	0.041	0.0098					1.02	0.0330
rs7871944	120338164	G	0.175	0.0078	0.173	5.01E-06	0.041	0.0092					1.02	0.0326
rs34789583	120345403	A	0.169	0.0100	0.172	5.77E-06	0.039	0.0136					1.02	0.0271
rs35940453	120348179	T	0.170	0.0096	0.172	6.00E-06	0.039	0.0144					1.02	0.0280
rs7857333	120353610	A	0.167	0.0111	0.171	6.72E-06	0.040	0.0113					1.02	0.0295
rs13299033	120355598	T	0.170	0.0096	0.172	5.71E-06	0.039	0.0143					1.02	0.0269
rs35199804	120358663	T	0.170	0.0095	0.172	5.82E-06	0.039	0.0140					1.02	0.0236
rs28693508	120361418	G			-0.062	0.0207	-0.062	2.99E-08						
rs1252008	120362838	C			-0.051	0.0439	-0.054	3.97E-07						
rs9408901	120364851	G			-0.051	0.0471	-0.054	4.24E-07						
rs10759926	120366225	C			-0.061	0.0235	-0.063	2.10E-08						

rs10983712	120368736	C	-0.062	0.0210	-0.062	2.49E-08
rs7047007	120371534	C	-0.051	0.0439	-0.054	5.22E-07
rs9299254	120374395	G	-0.051	0.0443	-0.054	5.04E-07
rs1013985	120374908	C	-0.051	0.0448	-0.054	5.08E-07
rs7038943	120377178	C	-0.061	0.0225	-0.063	2.02E-08
rs1928295	120378483	C	-0.051	0.0454	-0.054	3.98E-07
rs9408902	120378964	G	-0.051	0.0460	-0.054	3.97E-07
rs9299255	120380486	C	-0.051	0.0460	-0.054	4.02E-07
rs55654527	120380642	A	-0.060	0.0243	-0.063	1.92E-08
rs16905901	120381503	A	-0.108	0.0059	-0.084	3.10E-07
rs9409189	120382927	C	-0.050	0.0499	-0.054	4.53E-07
rs10116193	120385563	G	-0.060	0.0248	-0.063	2.17E-08
rs10116226	120385645	G	-0.060	0.0244	-0.062	2.29E-08
9:120386056_TA_T	120386056	T	-0.092	0.0172	-0.084	2.46E-07
rs10983719	120386402	A	-0.061	0.0232	-0.062	3.07E-08
rs716569	120387017	C	-0.109	0.0054	-0.084	2.82E-07
rs10983720	120387487	T	-0.060	0.0248	-0.063	2.17E-08
rs10818054	120388017	C	-0.060	0.0249	-0.062	2.22E-08
rs4443733	120389052	C	-0.051	0.0472	-0.054	4.27E-07
rs76002713	120393806	A	-0.175	0.0227	-0.146	4.75E-06
rs149607570	120394427	CGT	-0.072	0.0075	-0.062	2.54E-08
rs55823440	120396391	G			-0.052	1.56E-06
rs78417933	120397878	G	-0.142	0.0054	-0.089	2.86E-05
rs10759928	120398403	G			-0.053	6.19E-07
rs7032255	120400087	A	-0.060	0.0249	-0.062	2.72E-08
rs757526964	120400769	G	-0.106	0.0073	-0.080	1.14E-06
rs55928579	120402514	C	-0.108	0.0061	-0.082	6.59E-07
rs10119785	120403579	G	-0.111	0.0045	-0.085	2.20E-07
rs12001083	120404878	T	-0.053	0.0491	-0.062	3.82E-08
rs957512	120405705	C	-0.054	0.0476	-0.062	4.50E-08

rs12001662	120406009	T			-0.053	7.68E-07				
rs12348421	120410319	T	-0.087	0.0245	-0.073	6.09E-06				
rs10123561	120411867	T	-0.089	0.0047	-0.055	2.90E-05				
rs28671379	120412067	C	-0.088	0.0050	-0.055	2.80E-05				
rs928052	120414212	G					-0.021	0.0067	0.98	2.24E-05
rs13293271	120414769	A					-0.021	0.0070	0.98	2.36E-05
rs1887905	120417120	C					-0.023	0.0044	0.98	1.61E-05
rs12236328	120417648	A					-0.022	0.0050	0.98	1.68E-05
rs913615	120418118	C					-0.022	0.0052	0.98	1.72E-05
rs751232126	120418291	AC					-0.023	0.0044	0.98	1.41E-05
rs745507	120418759	C					0.074	9.48E-06	1.03	0.0024
rs4481681	120420226	A					-0.022	0.0055	0.98	1.88E-05
rs79825568	120420542	A					-0.023	0.0046	0.98	1.46E-05
rs10818063	120421431	G					-0.022	0.0047	0.98	1.50E-05
rs71497003	120421608	C					-0.022	0.0047	0.98	1.50E-05
rs36163427	120421796	C					-0.022	0.0057	0.98	1.57E-05
rs1928296	120423587	T					-0.022	0.0061	0.98	2.09E-05
rs1329068	120423703	G					-0.022	0.0058	0.98	1.99E-05
rs1410852	120424908	G					-0.022	0.0058	0.98	1.92E-05
rs11788318	120426508	G					-0.022	0.0060	0.98	2.37E-05
rs10818064	120426703	G					-0.022	0.0060	0.98	2.37E-05
rs10983741	120427319	C					-0.022	0.0060	0.98	2.49E-05
